# Giant pit craters on the modern seafloor above magma-induced hydrothermal vent complexes of Scotia Sea, offshore Antarctica

**DOI:** 10.1038/s41598-025-85899-y

**Published:** 2025-01-24

**Authors:** L. Somoza, K. M. Andreassen, A. Plaza-Faverola, T. Medialdea, J. Gafeira, F. J. González, G. R. Dickens

**Affiliations:** 1 IGME-CSIC Marine Geology Resources and Extreme Environments Group, Geological Survey of Spain, Madrid, 28005 Spain; 2 iC3-Centre for ice, Cryosphere, Carbon and Climate, Department of Geosciences, UiT The Arctic University of Norway, Tromsø, 9037 Norway; 3 BGS-British Geological Survey, Edinburgh, Scotland, UK; 4 Department of Geology, School of Natural Sciences, Trinity College Dublin, Dublin, Ireland

**Keywords:** Geodynamics, Geophysics, Tectonics, Volcanology, Climate-change impacts

## Abstract

Massive injection of ^13^C depleted carbon to the ocean and atmosphere coincided with major environmental upheaval multiple times in the geological record. For several events, the source of carbon has been attributed to explosive venting of gas produced when magmatic sills intruded organic-rich sediment. The concept mostly derives from studies of a few ancient sedimentary basins with numerous hydrothermal vent complexes (HTVCs) where craters appear to have formed across large areas of the seafloor at the same time, but good examples remain rare in strata younger than the Early Eocene. We present geophysical data documenting at least 150 large (km-scale) craters on the modern seafloor across ∼148,000 km^2^ of Scan Basin in the southern Scotia Sea, a remote region offshore Antarctica. Seismic and bathymetric information reveals the craters relate to vertical fluid pipes extending above dome-shaped forced folds and saucer-shaped igneous sills. Presumably, magmatic intrusions deform overlying sediment and produce thermogenic gas, where buoyant hydrothermal fluids migrate upwards from sill flanks through V-shaped gas chimneys to the seafloor. Fluid expulsion, driven by excess pore pressure, enhances vertical conduits and creates collapse structures on the seafloor. Age estimates for sill emplacement and crater formation come from correlations of seismic reflectors with bore hole data collected on IODP Expedition 382. Sills intruded into sediment at least two times, first about 12–13 Ma (Middle Miocene), which occurred with deep intrusions of stacked composite sills, and once about 0.9 Ma and associated with volcanism along Discovery Bank, which may have reactivated previous fluid venting. Crater reactivation has occurred since 0.9 Ma, although probably episodically. Importantly, at present-day, numerous craters related to sills and fluid pipes populate the seafloor above a young sedimentary basin, and the ocean and atmosphere are receiving massive quantities of ^13^C depleted carbon. The two phenomena are unrelated but, with changes in global climate and sedimentation, the craters could be filled simultaneously and give an impression in the rock record of rapid and coeval formation coincident with carbon emission. Interpretations of ancient HTVCs and their significance to global carbon cycling needs revision with consideration of modern seafloor regions with HTVCs, notably Scan Basin.

**Supplementary Information:**

The online version contains supplementary material available at 10.1038/s41598-025-85899-y.

## Introduction

Prominent and globally recognized negative carbon isotope excursions (CIEs) coincided with rapid global warming and environmental change several times during Earth’s history. These events, perhaps best exemplified by the Paleocene-Eocene Thermal Maximum (PETM; ca. 55.9 Ma)^1–4^ and the Toarcian Ocean Anoxic Event (TOAE; ca. 183 Ma)^5–8^ provide a crucial perspective for appreciating our future, because the CIEs signal massive release of ^13^C-depleted carbon (2000-12,000 gigatonnes, Gt) to the ocean and atmosphere^2,7,8^. Although we know how modern society can drive this through fossil fuel extraction and combustion, a satisfactory mechanism for past carbon injection events elusively lies at the forefront of geoscience research.

The large magnitude and rapid timing of CIEs across the PETM and TOAE necessarily implies a low ^13^C/^12^C ratio for the source of carbon, so many works invoke input and oxidation of CH_4_^1,7,8^. However, methane could derive from reservoirs on Earth’s shallow surface and as a feedback to environmental change (e.g., peat, permafrost, or gas hydrates) or, alternatively, through processes external to Earth’s surface, and as a forcing for environmental change (e.g., comets or contact magmatism).

Intrusion of magma into organic rich strata^9,10,11^ and release of gas from hydrothermal vent complexes (HTVCs) presents one explanation^12,13,^. At a basic level, an individual HTVC comprises a crater (1–2 km wide) on the seafloor with an underlying fluid conduit extending through sedimentary layers and connected to an igneous sill intrusion^14–16^. Outcrops and seismic reflection profiles show that, within the geological record, numerous HTVCs can terminate at a horizon across a moderately sized (> 100,000 km^2^) sedimentary basin^17–19^. Outstanding examples come from the Vøring and Møre basins (North Sea) and in the Karoo Basin (South Africa), which indeed have been linked to the PETM^18^ and TOAE^19^ respectively. HTVCs presumably represent transport and release of CH_4_ and CO_2_ generated at depth by the heating of sedimentary organic matter within contact aureoles around sills^20–22^. Based on numerical modelling^23,24^, hundreds of HTVCs might produce and release 5000 to 10,000 Gt of^13^C depleted carbon as CH_4_ and CO_2_, a mass that could explain carbon cycle anomalies across the PETM^2,6^ and TOAE^7,8^ However, such modelling makes key assumptions concerning sedimentary volumes affected by intrusive sills and rates of gas generation^23^. Indeed, production of 5000 Gt of carbon in a geological instant (< 20 kyr in the case of the PETM) invokes “catastrophism”, as this equates to the total pre-industrial mass of carbon stored in conventional oil and natural gas reservoirs across the globe^24^. Links between HTVCs in sedimentary basins and global carbon cycling thus remain highly debated, including in part because modern examples generally were not known.

**Fig. 1:**
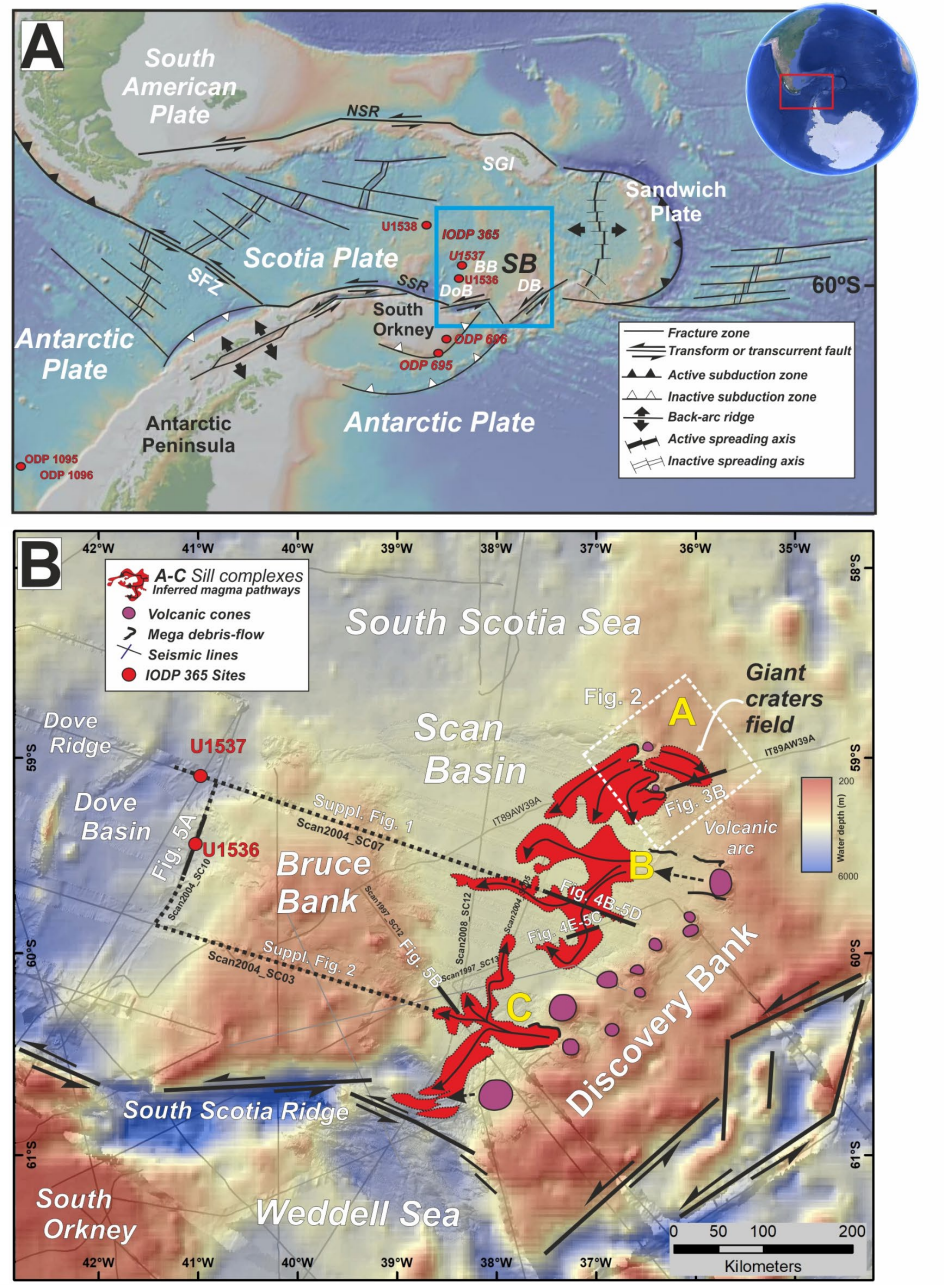
Overview of the study area. (**A**) Map showing plate boundaries, tectonic structures, bathymetry and key features surrounding Scan Basin (SB). BB: Bruce Bank, DB: Discovery Bank, DoB: Dove basin, SFZ: Shackleton Fault Zone, SSR: South Scotia Ridge, SGI: South Georgia Islands. Yellow dots show the location of ODP and IODP sites. Blue box shows the study area of lower panel. (**B**) Map of Scan Basin located between Bruce Bank and Discovery Bank and north of South Scotia Ridge that highlights significant subsurface structures. Red: areas with mapped subsurface sills based on the isochrone map to the top of the sill intrusions. Black lines: inferred transport magma flow directions. Yellow letters (A, B and C): sill systems. Magenta colour marks the volcanic cones along the Discovery Bank. Black bold lines mark the localization of the seismic lines show in Figs. 3B and 4A and E. White box show the location of the field of giant craters covered with high-resolution bathymetry shown in Fig. 2. V1 and V2 are volcanic cones shown in Figs. 2 and 8A associated with the field A of pit craters.

We report and discuss here numerous giant craters and pockmarks across three areal extensive “fields” within 148,000 km^2^ of the modern seafloor of Scotia Sea, a remote and largely unexplored region between Antarctica and South America (Fig. 1). Because fluid conduits connect the seafloor craters to saucer-shaped sills that have intruded sediment of Scan Basin, we consider this area a modern example of fossil HTVC systems in the geological record that have been linked to global warming and environmental change in Earth´s history^1^. We integrate interpretations of seismic and multibeam bathymetry data and place this information with ages determined from drilling results of International Ocean Discovery Program (IODP) Expedition 382^25^ to constrain the onset and activity of craters and related magmatic intrusions (Fig. 1). We suggest modern craters in Scan Basin reflect relatively long-lived processes, which leads to alternative interpretations for the timing and significance of HTVCs in the geological record.

## Geographical and geological setting

Scan Basin lies between Bruce Bank and Discovery Bank in the central and southern Scotia Sea (Fig. 1). The basin serves as a main deep-water gateway allowing flow of Antarctic-sourced waters into the Circumpolar Antarctic Current as well as northwards into the Atlantic Ocean^26,27^. The tectonic history of the region is critical to understanding the opening of Drake Passage, the development of the Antarctic Circumpolar current, and thermal isolation of Antarctica^28^.

Beyond being a paleoceanographic target, a truly fascinating find on expeditions to Scan Basin carried out in 1997, 2000, 2004 and 2008 onboard R/V *Hespérides* as part of the SCAN Project^26–29^ has been discovery of a modern HTVC system. Buried sill complexes with associated fluid conduits and seafloor craters span large areas of southeastern Scan Basin (Fig. 1). Seismic profiles across the area also display bottom simulating reflectors (BSRs), one set which likely signifies the base of the gas hydrate stability zone (BGHSZ) and an interface between overlying gas hydrate and underlying free gas occupying sediment pore space^29^. To appreciate this concept and other aspects of sedimentary sequences, we note four characteristics across the region: (1) low water-bottom temperatures, as low as -0.5 ºC at 2000–3000 m depth^29^; (2) strong bottom-currents and high sediment discharge from melting icebergs, which when combined, lead to giant contourite drifts and sedimentary waves^26,30^; (3) significant variations in geothermal gradients because of complex tectonics^31^ and (4) giant mass transport deposits^32^ (Fig. 1 and Supplementary Figure S3).

## Results

**Fig. 2:**
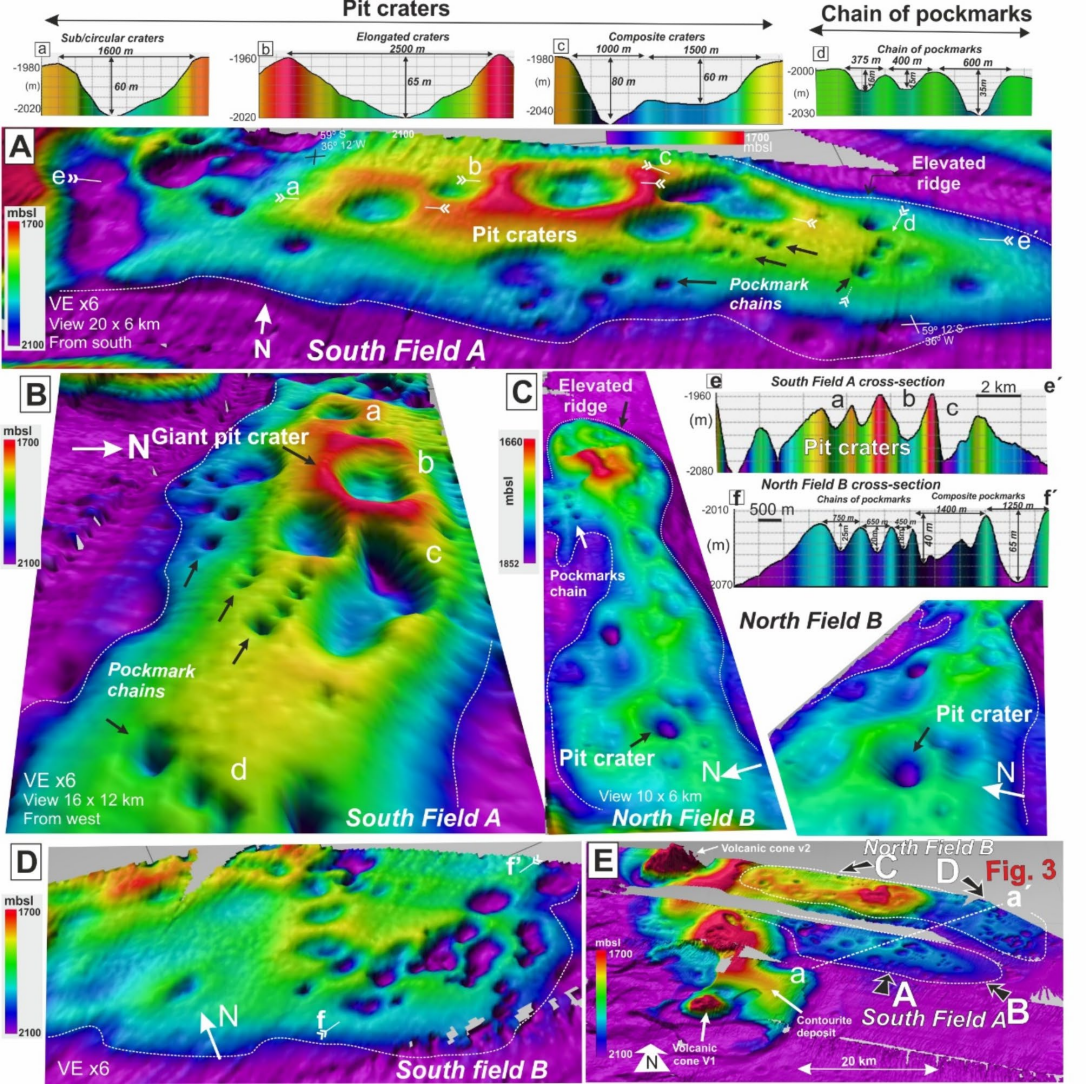
High-resolution bathymetric images of the seafloor craters and pockmarks. (**A**) and (**B**) show 3D images from the South Field A and (**C**) and (**D**) show 3D images from the North Field B. a-f insets show the profiles and dimensions (width and depth) along the craters. (**E**)-Overview of the study area. Arrows show orientation of the 3D views (**A**–**D**). Dotted white line indicates location of 2-D seismic line shown in Fig. 3. V1 and V2 are volcanic cones associated with the field of pit craters. Information of the vulcanism in the Supplementary Figure S3. Note that colour scale of the bathymetry is different for individual panels.

High-resolution bathymetry reveals over 150 giant craters and pockmarks covering at least 8,760 km^2^ of the seafloor in the area between Scan Basin and Discovery Bank (Fig. 2A-D). With available information, the craters are located between 1,750 and 2,220 m water depth across two “fields” (A and B) located above the eastern part of the sill complex A (Fig. 1B). This area is characterised also by contourite drifts, large submarine slides and prominent volcanic cones^31^ (Fig. 2E and Supplementary Figure S3). For both fields, seismic profiles highlight relationships between craters, pockmarks and sill intrusions, the latter which extend across ∼148,000 km^2^ beneath the seafloor of Scan Basin (Fig. 1B).

### Seafloor morphology of giant pit craters and pockmark-chain fields

The term ‘pit crater’ is used here to refer to sub-circular seabed depressions 1–2 km wide and some tens of meters in depth formed in unlithified tills and marine sediments^33,34^. For smaller seabed depressions, we use the term ‘pockmark’, and classify these according to their diameters as giant pockmarks 100-1,000 m, standard pockmarks 5–100 m^35,38^ or unit pockmarks < 5 m^37^.

**Fig. 3:**
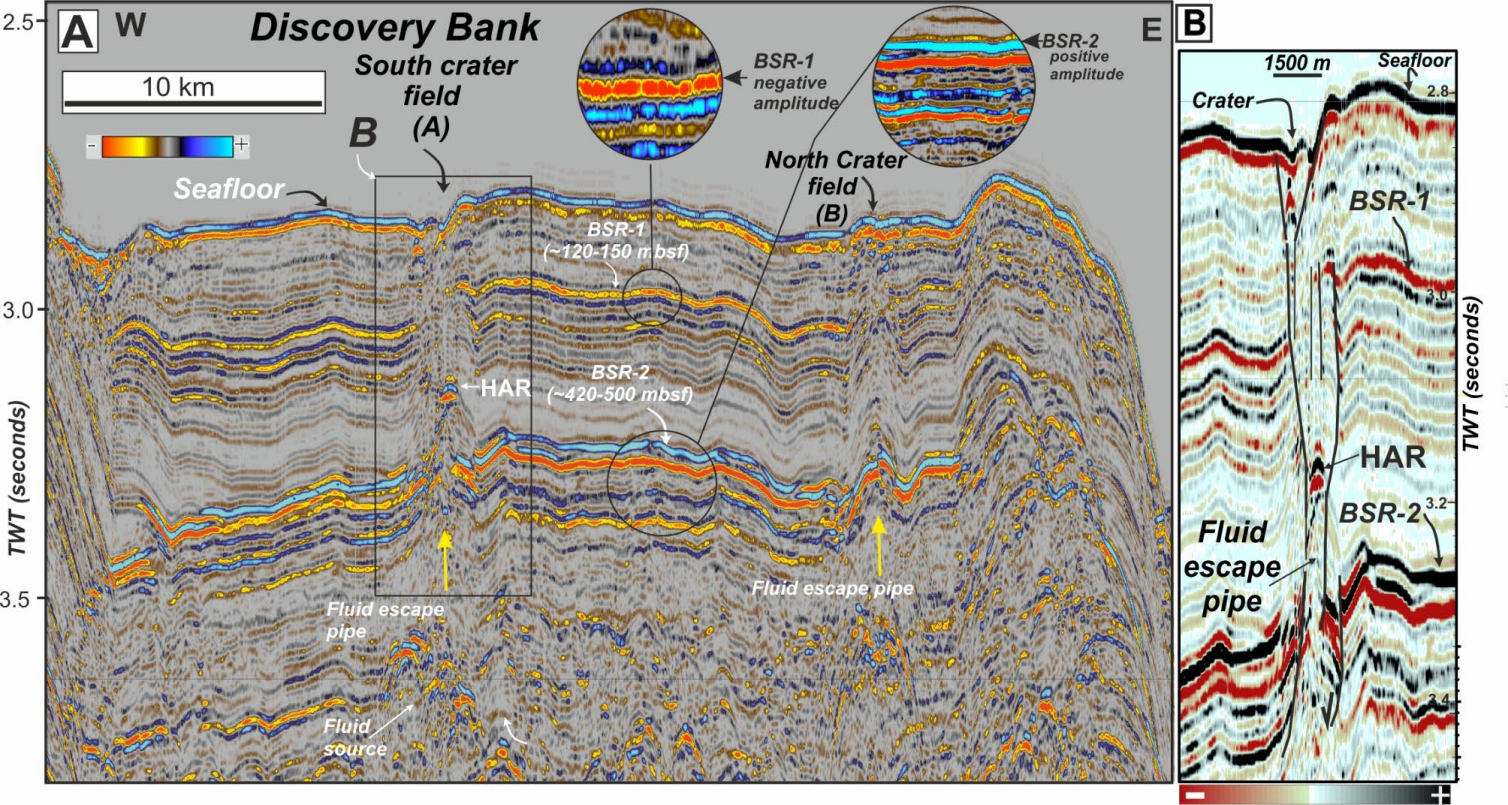
High-resolution seismic image of the subsurface beneath seafloor craters (**A**) Seismic line crossing the field of craters in a SW-NE direction as shown Fig. 2E. Circle insets show details of the reflective polarity of the main bottom simulating reflectors (BSRs) identified at regional scale in the study area. BSR-1 is characterized by a reverse polarity with respect to the seafloor polarity whereas BSR-2 has the same polarity as the seafloor. Both are breached and collapsed by fluid escape pipes beneath the crater fields A and B. (**B**) Detail of subsurface beneath the craters showing a fluid escape pipe breaching the two BSRs. HAR: High Amplitude Reflector within the pipes.

A variety of seabed pit craters and giant pockmarks are identified in Field A, which is associated with a ring of volcanoes along Discovery Bank to the east, including two outcropping volcanic cones (Figs. 1A and 2F). Craters and pockmarks align along general ENE-SWS trends with depression depths (relative to surrounding seafloor) ranging from 20 to 80 m (Fig. 2). A seismic line crossing the two fields of seafloor craters (a-á in Fig. 2E). show vertical chaotic zones (VCZs)^38^ beneath the pit craters, characterised by acoustic signal significantly attenuated with respect to that in surrounding strata and interpreted as fluid migration pipes or chimneys^39,40^ (Fig. 3A).

Pit craters range from nearly circular to elliptical, and sometimes seem spatially linked, including twin-like craters. For elongated features, the eccentricity index (short axis/long axis) ranges from 0.35 to 0.88, with the reported diameter understood as the greater axis length. The largest craters have long-axis lengths ranging from 1.6 to 2.5 km, and depths between 60 and 80 m. Importantly, they generally lie on bathymetric highs, which broadly align along axes of mounded structures (Field A in Fig. 2A-B and Field B in Fig. 2C-D) and the presence of antiforms (`forced-folds´) and sub-subsurface sills inferred from seismic profiles^32^. The axis of the northern field A is composed by four major pit craters ranging from 1,600 to 2,500 m in diameter (Fig. 2A). The largest pit crater in Field A (`b´ in Fig. 2A) is characterised by a rim of sediment suggesting ejection of solid material around a seafloor cavity. Surrounding these major pit craters, pockmarks riddle the seafloor with diameters ranging from 350 to 750 m and depths of 20–40 m (Fig. 2A-B). These `standard´ pockmarks can form chains of 3–4 features next to each other as in Field A (Fig. 2A-B), or exist randomly distributed as in Field B (Fig. 2C-D). Both pit craters and standard pockmarks are characterised by steep slopes, generally 15º to 26º but with gradients up to 45º on their flanks (Fig. 2A).

### Subseafloor fluid pipes breaching seismic horizons beneath pit craters

The combination of multibeam bathymetry (Fig. 2E) and 2-D multichannel seismic lines (Fig. 3) shows the largest craters of Fields A and B lie above vertical chaotic zones (VCZs)^38^ (Fig. 3A). These VCZs, characterized on seismic data by columnar zones of reduced reflection continuity and often termed in the literature as fluid escape pipes, chimneys or gas chimneys^39,40^, are interpreted as conduits where fluids (including gas) migrate upward though strata, perhaps brecciating sediment through hydro-fracturing^41^. Interestingly and importantly, for Scan Basin, these VCZs breach two high-amplitude regionally extensive sub-horizontal reflectors^42,43^. These bottom simulating reflectors (BSRs) have different signatures and probably represent two different processes affecting sonic velocity of sub-seafloor sediment in Scan Basin, similar to other better studied areas, particularly northern subpolar regions^44^ (Fig. 3A-B).

The upper BSR-1 exists at around 120–150 ms two-way time (TWT) below the seabed (Fig. 3). It is characterised by reversed amplitude (or negative polarity; insets in Fig. 3A), which signifies a drop in sonic velocity. Likely, this represents the BGHSZ, consistent with calculations assuming seawater chemistry, 100% methane gas, bottom water temperatures and regional geothermal gradients^28^. BSR-1 thus marks the boundary between overlying sediments that host gas hydrate and underlying sediments that host free-gas^44^.

The lower BSR-2 lies at 430–500 ms TWT below the modern seabed (Fig. 3). Its discordant nature and enhanced seismic amplitude (or positive polarity) is consistent with a major increase in sonic velocity. A diagenetic transition of bio-silica components, where opal A converts to crystoballite and tridymite (opal CT) with increased burial depth and time provides one good explanation^45,46^. Certainly, in sedimentary sequences around Antarctica, which contain abundant bio-silica, the opal A/CT transition has been identified as an important process affecting sediment composition and physical property measurements^47–49^. However, data from the most proximal ODP and IODP sites to Scan Basin (Fig. 1A) allow for alternate views of this reflector. At ODP Site 696^50^, located off South Orkney, a clear diagenetic transition between opal A and opal-CT was reported at 520–530 mbsf, and this caused an increase in P-wave velocity up to 3000 m/s and a decrease in porosity from 75 to 44%. On the other hand, at IODP Site U1536 (Fig. 1A), located in Dove Basin, a sharp increase in P-wave logger velocity (PWL from 1600 to > 2000 m/s) represents a downhole change to fully lithified mudstone^25^. However, in deeper sediment at IODP Site U1536, core recovery was especially poor (in some cases 3%), and dissolved silica concentrations show a marked drop consistent with silica removal^25^. The cause for the reflector must be regarded as uncertain, although it definitely represents a relatively sharp transition to more lithified sediment.

Horizon BSR-2 marks a regionally important lithological boundary (Supplementary Figures S1 and S2). Because it represents the top of harder rock, the convex upwards deformation and high-amplitude reflectors (HARs) identified at the intersection between BSR-2 and vertical fluid pipes are interpreted as hard rock fragments displaced by upward migrating fluids (Fig. 3). This may hint at the strength of rising fluids related to the formation of pit craters on the seabed.

### Sill intrusions and forced folds

**Fig. 4:**
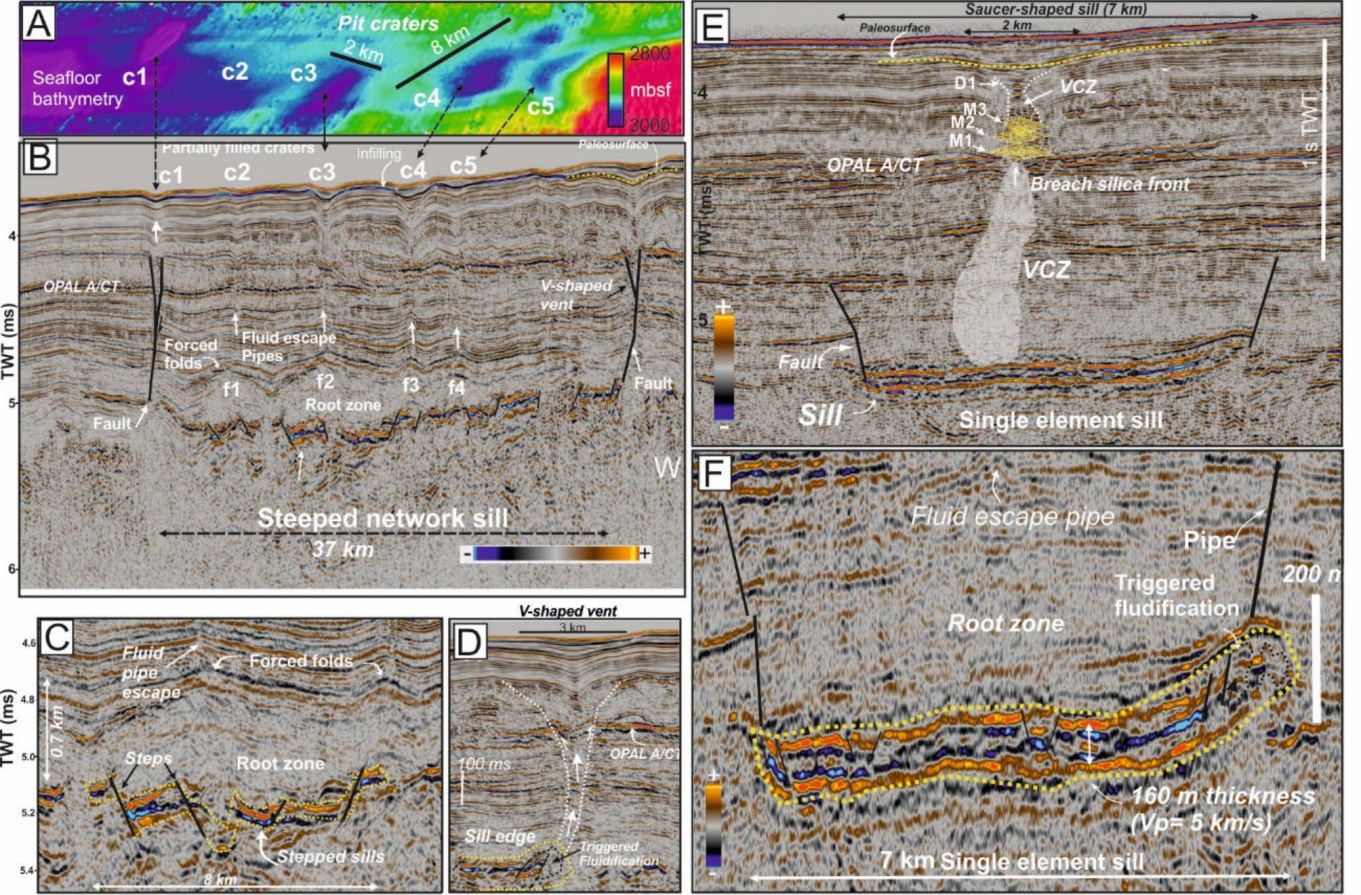
Seismic characteristics of magma-induced hydrothermal buried systems as the source of seafloor craters. (**A**) Multibeam bathymetry mosaic along the seismic line displayed in (**B**) showing elongated sea-floor craters labelled as 1–4. (**B**) Seismic line showing the complete hydrothermal buried system formed above a complex of interconnected (steeped) sills. Seabed craters are connected throughout fluid pipes above the forced folds formed by the intrusion of the steeped sills. (**C**) Detail of forced folds formed above the reservoir as consequence of generation of thermogenic methane. (**D**) Detail of V-shaped fluid pipe at the edge of the sill complex. (**E**) Seismic image of the hydrothermal buried system formed above a single saucer shaped sill. A gas chimney is formed in the centre of the sill breaching the opal diagenetic layer. (**F**) Detail of the seismic expression of a single saucer-shaped sill, 7 km long and 160 m-thick, characterized by two strong positive reflections surrounding a negative reflection. Location of seismic lines is shown in Fig. 1.

**Fig. 5:**
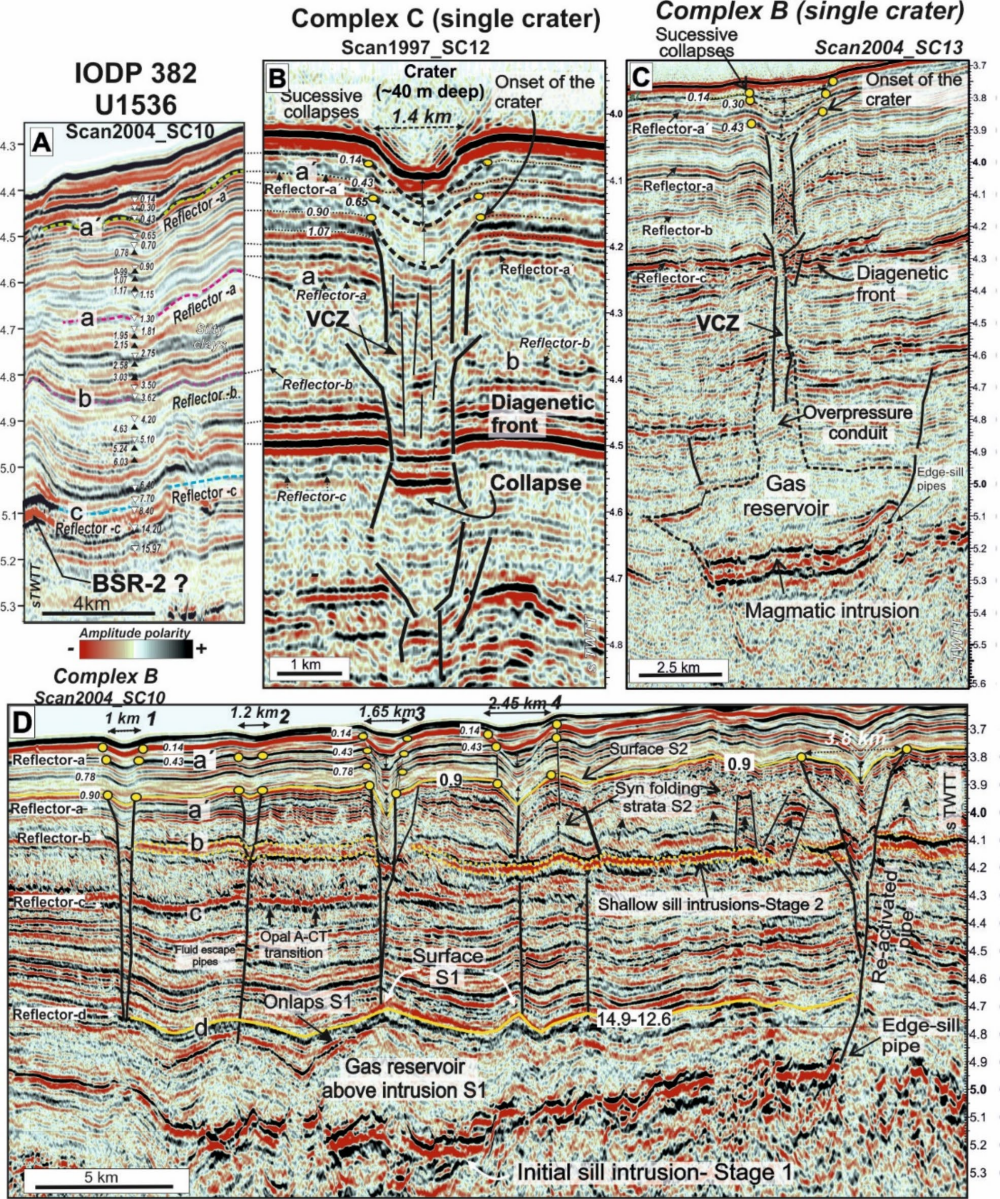
Constraint ages of the formation of the craters based on the correlation with IODP expedition 382 site Site U1536. (**A**) Age constraints over seismic background based on biostratigraphic and paleomagnetic ages^57^. (**B**) Seismic record of a single crater from complex C (**C**) Seismic record of a single crater from complex B. (**D**) Seismic record of the sequence of craters from complex B. See Fig. 1B for location. Yellow dots are the inflection points of the reflectors interpreted as marking the events of collapses of the craters (1–4) shown in Fig. 4A. VCZ: Vertical Chaotic Zone. S1 and S2 are the paleosurfaces above the forced folds marking the timing of sill emplacements according Magee et al.^55^. Numbers are age in My ties with the U1536 Site^25^.

Prominent seismic reflections characterized by high amplitude and abrupt terminations appear as sub-horizontal sheets that intrude into deep sedimentary layers of Scan Basin (Fig. 4). These are interpreted as igneous sills^9,10^. Using available 2-D multichannel seismic lines (Fig. 1B), the sills we document here compose part of a magmatic complex spanning at least ∼148,000 km^2^. Between the deep subsurface of Scan Basin and Discovery Bank, three main sill complexes labelled, from north to south, as A, B and C, have been identified on seismic lines (Fig. 1B). We used a combination of seismic horizons to the top of sills (map-view) with seismic section (cross-section view) to map sill morphologies. The distribution of sills in planar view (Fig. 1B) and the elements identified on seismic sections suggest that each sill complex was derived from three distinct magma feeders located along the Discovery Bank volcanic arc with a flow down dipping to the west (Fig. 1B). Sill intrusion emplacement took places at considerable depth within the Scan Basin (5,000–5,500 ms TWT, or between ca. 1 and 1.5 km below seabed, assuming a P-wave velocity of v = 2.5 km s^− 1^ (Fig. 1B). Seismic expressions of the sills show diverse morphologies from element networks to single elements, at least as with available 2-D seismic reflection highlighting the deep strata of Scan Basin^51,52^ (Figs. 4 and 5). Here we use the term element to generally describe the building blocks of sills without attempt to infer their 3-D geometries or emplacement geometries^52^.

Thus, seismic sections suggest two end-member types of sheet-like igneous elements^9,52^: extensive networks composed of several connected sill elements with step-like geometry (Fig. 4B and C), and single element sills (Fig. 4E and F). Sill networks consists of igneous bodies up to 37 km long and 110–140 m thick (assuming a P-wave velocity of 5500 m/s for mafic sills^52^), emplaced at depths of ~ 1000–1500 m below the seafloor (i.e., at 5000–5500 ms TWT), and composed of connected elements presumably sourced from the same parent magma body, albeit perhaps through several magma pulses^51,52^ (Fig. 4B). By contrast, single element sills consist of an isolated element, 5–7 km in length and 130–150 m in thickness (Fig. 4E and F). These “saucer-shaped sills” typically consist of a flat inner base connected to inclined outer edges, and normally appear as second order elements at the end of the inferred magma flow direction^52^ (Fig. 1B).

Above sill networks, interconnected forced folds^53,54^ are recognised in regional stratigraphy (f1 to f4 in Fig. 4B). In the most prominent example (Figs. 4B and 5D crossing Complex B), and from west to east, buried folds have axes 6.0 (f1-f2), 5.0 (f2-f3) and 4.3 km (f3-f4) apart, with structural relief of nominally 580, 760 and 590 m between (assuming P-wave velocity of v = 2.5 km s^− 1^, Fig. 4C). Such folds are similar to ‘compound forced folds’ described from strata in the North Atlantic and associated with polyphase intrusive events^55^. The acoustic blanking zone located between the top of the sills and within strata with forced-fold antiforms might indicate accumulation of fluids in pore space, particularly including CH_4_^54^. Above the forced folds, ~ 1.5 km long vertical pipes connect to semi-filled craters on the seafloor (c1 to c5 in Fig. 4B) (Fig. 4B). The seafloor expression of these features and their relation to underlying sills are very similar to that shared from deeply buried Paleogene strata of the More and Vøring basins, west of Norway^56^. Notably and especially above single element sills in Scan Basin (Fig. 4E), fluid migration appears acoustically as VCZs suggesting continuous and current re-utilization of fluid flow pathway(s)^38^.

### Chronostratigraphic constraints

The timing of sill intrusion and seabed pit crater formation can be bracketed through correlations of seismic reflectors from SCAN project cruises^57^ with age dates from the upper sedimentary record of Scotia Sea sites drilled by IODP Expedition 382^25^ (Fig. 1A). Seismic units drilled at Sites U1356 and U1537 located in Dove Basin can be correlated to those in Scan Basin using seismic profiles Scan2004_SC10, Scan2004_SC07 and Scan2004_SC03 (Figs. 1B and 5, and Supplementary Figures S1 and S2). The drill sites also have paleomagnetic and biostratigraphic data that render a well-developed age model^58^. The drilled sedimentary units have been correlated to four basin-wide reflectors, named (from uppermost to lowermost), a’, a, b and c (Fig. 5A), which somewhat correspond to major changes in the lithostratigraphy of Site 1536^25^. Unit I (seafloor to ~ 250 mbsf) consists of interbedded diatom oozes and silty clays (and well-defined reflectors) deposited from the early Pliocene to present-day. Unit II (~ 250–550 mbsf) is mainly composed of silty clay and variable amounts of bio-silica deposited from the Late Miocene through the early Pliocene, and with lithification increasing downhole. Unit III (~ 550 mbsf to the base of Hole U1536E at 643 mbsf) consists of semi- to fully lithified mudstone, some with biosilica and beds of limestone. The strong Reflector c marks the Unit II/Unit III boundary and a sharp increase in p-wave velocity and other physical properties^25^. This horizon has been interpreted as the opal A/opal CT diagenetic transition around Antarctica^28,47,49^ and in other areas^46^, although this is not fully supported with the poor and discontinuous drilling in the region (above). With this information, approximate ages for the reflectors are: Reflector-a´, 0.4 Ma (Middle Pleistocene); Reflector-a, 1.7 Ma (Early Pleistocene); Reflector-b, 4.5/3.7 Ma (Pliocene); Reflector-c, < 8.4 Ma (Late Miocene)^57^.

### The timing of intrusive events in scan Basin

We use available seismic stratigraphy to constrain the timing of sill intrusions. Onlaps of sediment layers (paleo-surfaces) onto the intrusion-related forced folds can be used as indicators to estimate the timing of magma emplacement^55^. Two main paleo-surfaces provide timing relationships (Fig. 5D). A lowermost S1 paleo-surface marks the top of compound forced folds at 4.7–4.8 s TWT (Fig. 5D). This S1 paleo-surface correlates with Reflector-d in Scan Basin^58^. Reflector-d represents the base of Unit IV (Middle-Early Miocene) described in Scan Basin with an estimated age of 14.6–12.6 Ma^58^ but below the depth of regional drilling to date. A stratigraphically higher S2 paleo-surface lies at 3.8–3.9 s TWT, and corresponds to the base of seabed pit craters identified within Scan Basin. With our correlation to the sediment sequences drilled by IODP Expedition 382^25^, the S2 paleo-surface marks an unlabeled reflector and a 0.9 Ma isochron.

### Pit crater timing and generation

Seismic-stratigraphic relationships between underlying stratigraphy and overlying pit craters allows us to estimate the onset and successive reactivation of the seafloor features^55^. Some reflections above the base of the pit craters appear thickened, onlap on the walls of fluid escape structures, or both (Fig. 5B-D). These are interpreted as bodies of unconsolidated sediment that accumulated within pit craters when they were exposed on the seafloor, and can be correlated to the stratigraphic column drilled on IODP Expedition 382^25,57^ (Fig. 5A).

The oldest buried surface expression of pit craters above sill network complexes B and C are located along a paleosurface between Reflector-a´ (1.30 Ma) and Reflector-a (0.43 Ma) (Fig. 5A). We estimate that the paleosurface marking the onset of pit craters in this region corresponds to about 0.9 Ma (Early Pleistocene) (Fig. 5B and D). Within the pit craters, thickened reflections interpreted as fill deposit occurred at approximately 0.65 Ma, 0.43 Ma (Reflector -a´), and ca. 0.14 Ma (Fig. 5B–D; Table 1). However, we do not know if sediment infilling across pit craters was coincident. However, the shallowest expression of sediment filling these craters, at apparently at 0.14 Ma, marks an age from which sediment has slowly accumulated until present-day, allowing the morphological expression of these craters identified on sea floor (Fig. 4A).

**Table 1:** Constraints on the age (Ma) of the formation and re-activation of craters from Complex A to C (from South to North, location in Fig. 2B) in the scan Basin based on the correlation with IODP Expedition 382 drilling sites U1536 and U1537.

Sites U1536-U1537 (59–60° S)	Complex C (60° 30ʹ S)	Complex B proximal (59° 30ʹ S)		Complex B distal (60° S)	Complex A (59° S)
Scan2004_SC10 (Fig. 5A)	Scan2004_SC03 (Fig. 5B)	Scan2004_SC07 (Fig. 5D)		Scan2004_SC13 (Fig. 5C)	ITA89AW39A (Fig. 3B)
Type of crater	Single	Single V-shaped at sill edge	Cluster V-shaped at middle	Single V-shaped	Crater fields A and B
Expression on seafloor	Semi-buried	Buried	Buried	Buried	Exposed morphologically (Fig. 2)
	**0.14 Ma**		**0.14 Ma**	**0.14 Ma**	**0.14 Ma**
			**0.30 Ma**	**0.30 Ma**	*0.30 Ma*
Reflector–a´	**0.43 Ma**		**0.43 Ma**	*0.43 Ma*	
			**0.78 Ma**		
Onset	*0.90 Ma*	*0.90 Ma*	*0.90 Ma*		
Reflector–a					

Italics indicates the onset of the craters.

Bold indicates the re-activation of the craters.

Seismic expressions of other craters, especially those linked to single element intrusions above sill complex B (Figs. 1B and 5C) consistently show a youngest age for coincident with Reflector-a´ (0.43 Ma, Mid Pleistocene). Above this reflector, interpreted fill deposits occurred at 0.30 and 0.14 Ma.

Apprarent time differences between crater formation and sediment filling (Fig. 5) suggests multiple magma pulses. Indeed, we suggest that single element sills represent second-order propagations from a first-order network, one with a flow direction from a presumed sources located along the Discovery Bank (Fig. 1B and Supplementary Figure S3). Coincident re-activation of seafloor craters formed above sill intrusions may provide a general explanation for multiple observations (Table 1).

Otherwise, pit craters of the fields A and B sourced from Complex A appear clearly on bathymetric maps (Figs. 1B and 2, and Supplementary Figure S3). From above, many of these craters formed before 0.30 Ma (and probably Ma before), and they have been active since 0.14 Ma (Late Pleistocene). An acknowledged lack of seabed samples, ROV images or high resolution echosounder data from this challenging and remote area does not allow us to assess whether giant pit craters of Scan Basin are presently active.

## Discussion

### Long-term HTVC system and re-utilisation of fluid flow pathways

A key issue for the modern HTVC system in Scan Basin and for past examples in sedimentary basins of the geological record is determining relationships between the age of magma emplacement, the age of vent formation, and the age of vent filling. For estimating the time of initial magma emplacement, we use the age of the folded and uplifted paleosurface induced by magmatic activity. This is similar to approaches for dating Paleogene and Lower Eocene sills in the NE Atlantic^55^. For the NE Atlantic, seismic reflections that onlap onto forced folds provide constraints on magmatic activity throughout the Palaeocene and Early Eocene. For Scan Basin, we have distinguished two main paleosurfaces (S1 and S2) interpreted as developing directly by intrusion-induced forced folds. Sediment sequences onlapping these paleosurfaces should give relative ages for magmatic intrusion. Correlation of these paleosurfaces with lithostratigraphic units drilled at IODP Sites U1357 and U1536 should further allow absolute age constraints of sill intrusions. Significant magmatic intrusions occurred at ca. 12.9 Ma (Middle Miocene) and at 0.9 ca. Ma (Early Pleistocene).

Magma intrusions into sediment of Scan Basin therefore took place at least twice. However, these times are not necessarily those of fluid venting, which in Scan Basin is represented by reflectors marking where and when pit craters were seafloor features^32^. Following this inference and chronostratigraphic constraints based on ties to IODP Expedition 382 data,^,^ we suggest major fluid venting and the first generation of pit craters began at 0.9 Ma (Early Pleistocene). As stressed above, though, sedimentary sequences within the pit craters hints at successive episodic re-activation followed by partial infilling through the Mid to Late Pleistocene (Fig. 5D). Besides accumulation of stratigraphic sequences within pit craters, fluid venting sourced from the sills also appears as a sequence of stack mounded structures that breach reflectors -c and -b (8.4–4.5/3.7 Ma, Late Miocene-Pliocene). The overall seismic relationships resemble those observed near volcanic vents located above a > 157 km^2^ late Cretaceous volcanic field in Great South Basin, offshore New Zealand^38^. Here, also authors suggested episodic re-use of fluid pathways for over 54 Ma^38^.

### Evolutionary model for sill emplacement and fluid venting

**Fig. 6:**
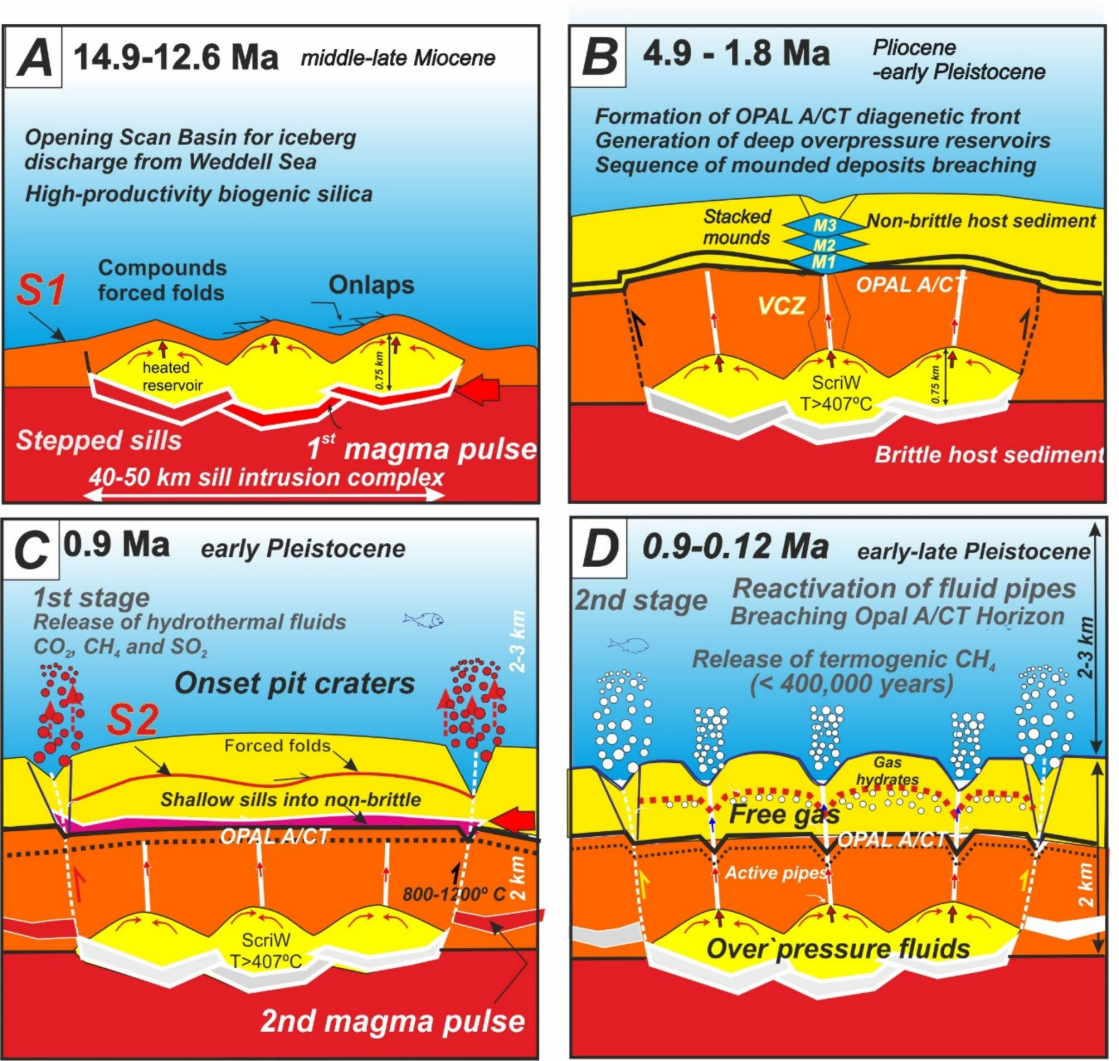
Proposal of evolutionary model of the sill-induced buried hydrothermal system. (**A**) Magma emplacement during the 1st magma pulse at ca. 14.9–12.6 Ma (middle-late Miocene) generated forced folds above sills by uplift deformation. Continuous release of thermogenic gas from cracking trapped beneath the forced fold-induced domesformed huge thermogenic reservoirs. (**B**) Opal diagenetic front sealed these reservoirs under conditions of supercritical waters within the buried hydrothermal system^60^. Focused breaching of the opal A/CT Horizon triggered stacked mounds by focused fluid venting above this horizon estimated to occur between horizon-b and -a (4.9–1.8 Ma, Late Miocene-Early Pliocene) according Pérez et al.^57^; (**C**) A second magma pulse at ca. 0.9 Ma triggered the fluid pipes to the surface forming the sequence of V-shaped craters and (**D**) Fluid migration sourced from the deep thermogenic reservoirs took at successive reactivation allowing to reach to the surface. Further explanation in the text.

To explain the overall distribution of sills, fluid conduits and craters within the context of documented seismic stratigraphy, we propose four stages for the evolution of Scan Basin HVTCs (Fig. 6).

A. An initial huge emplacement of magma at ca. 14.9–12.6 Ma (middle-late Miocene) and broadly coincident with Reflector-d reported in the Scan Basin^26^ (Fig. 6A). This first magmatic pulse at ca. 14.9 Ma might relate to the opening of Scan Basin and its connection with Weddell Sea because of eastward movement of the subducting arc to the east^58^. Such connection of Scotia Sea and Weddell Sea may have also allowed for increased circulation of large icebergs and sedimentation to the seafloor. Modelling fluid flow in sedimentary basins with sill intrusions shows that hydrothermal plumes initially form at outer edges of the intruded sheet sills^24^. This explains the preferential location of inferred faults at the edges of most sheet intrusions. Importantly, modelling gas generation around igneous sills in sedimentary basins further suggest that buoyant, methane-rich fluids might form above the inner sill from cracking of sedimentary organic carbon molecules, generating finger-like plumes that grow and coalesce above the sills^25^. Furthermore, analogue models for intrusion of shallow magma^59^ also show that this may result in growth of dome-shaped forced folds, and development of reservoirs for geothermal supercritical fluids^60,61^ in sedimentary layers low permeability. In the specific case of Scan Basin, increased sedimentation rate after connection to Weddell Sea^57^ may have favoured the sealing of thermogenic gas reservoirs above sill intrusions.
B. Formation of a physical property boundary between lithified lower sediment and unlithified upper sediment -- now represented by Reflector-c. This could be the opal A/opal CT transition, and further sealing of thermogenic gas reservoirs caused by sill intrusion. In any case, localized breaching caused sucessive stacking of mounded structures within conduits by fluid migration above this boundary from 4.9 Ma to 1.8 Ma (Pliocene to early Pleistocene) (Fig. 6B).
C. A second shallow magma pulse at 0.9 Ma (Middle Pleistocene) (Fig. 6C). During this stage, massive fluid venting occurred through reactivation of former Miocene deep reservoirs and re-utilization of these vertical pipes. This magma pulse might relate to volcanism developed along Discovery Bank. Basalts and subvolcanic dolerites were collected from volcanic cones proximal to the giant pit craters above the Complex sill C, located in the Discovery Bank (Figs. 2 and 8A and Supplementary Figure S3).
D. Further fluid venting from 0.9 Ma to 0.12 Ma (Middle to Late Pleistocene) (Fig. 6D) after emplacement of shallow magma. This is evidenced by multiple intervals of sediment infilling within the pit craters.

The above interpretation implies that Scan Basin HTVCs and their plumbing systems were utilized for fluid flow over ca. 15 Ma. Longstanding fluid flow pathways probably provided a return flux of organic carbon from source rocks to overlying reservoirs and ultimately the deep ocean. Studies of several fossil HTVC systems have also suggested they represent polyphase events and episodic re-use of fluid pathways. Good examples of this are reported from a late Cretaceous volcanic field in the Great South Basin, offshore New Zealand with inferred fluid migration extending over 54 Ma^38^, volcanic intrusions within Rockall Basin with inferred fluid migration extending for 15 Ma between the Early Paleocene and Middle Eocene^55^, and hydrocarbon fluid migration from buried magmatic bodies in the South China Sea that extend from ca. 35 Ma to present day^62^.

### Formation and infilling stages of pit craters

Pit craters are enigmatic, quasi-circular depressions observed on rocky (e.g., Earth and Mars) and icy (e.g., Enceladus) planetary bodies, as well as on asteroids^32^. These depressions do not reflect meteorite impacts. Instead, they are thought to be generated by overburden collapse into a subsurface cavity (e.g., created by dilational faulting) or a volumetrically depleted zone (e.g., an evacuated magma conduit)^32^.

**Fig. 7:**
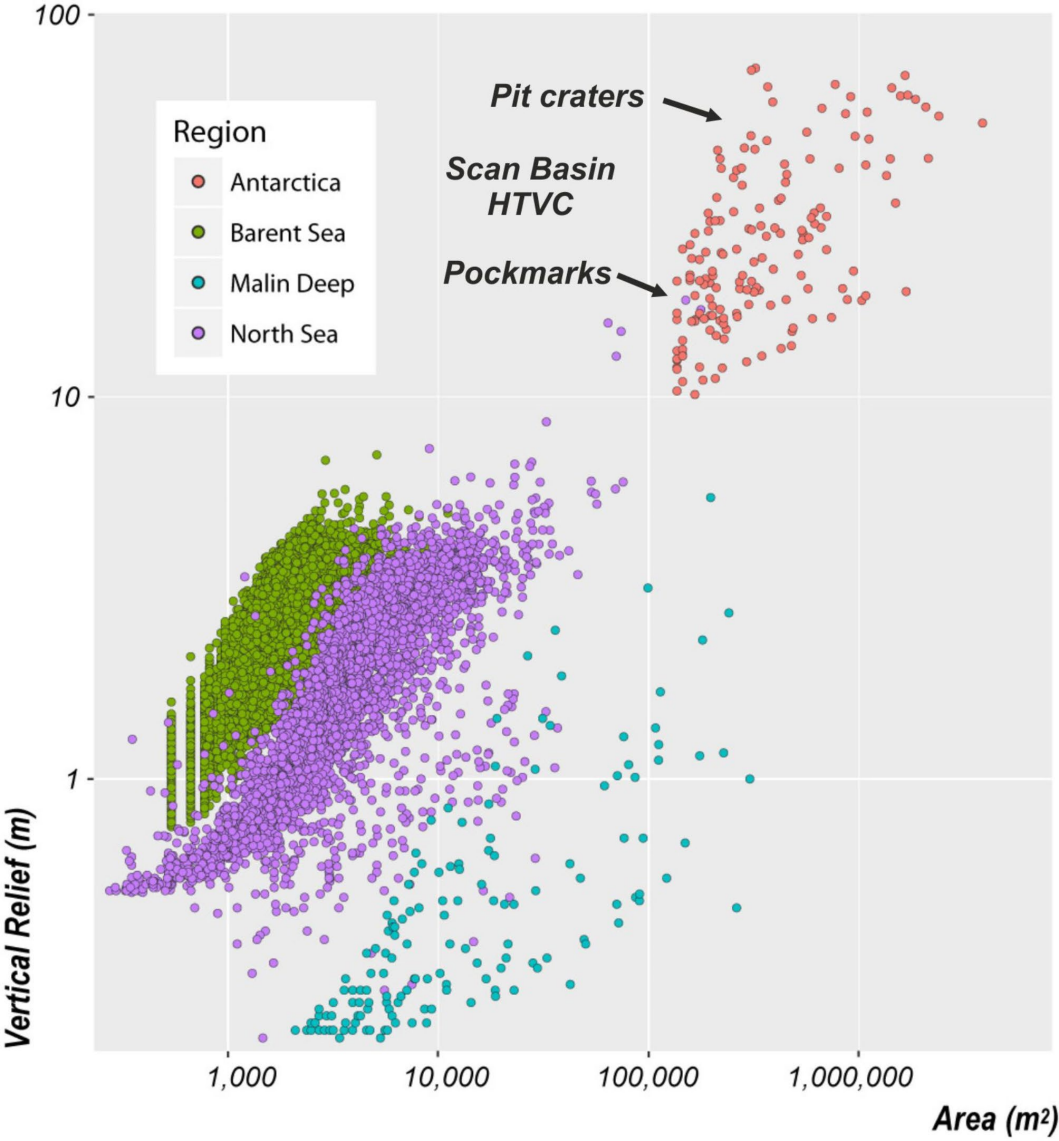
Comparison of giant craters in Antarctica with seafloor craters in other worldwide areas. Vertical Relief as a function of Area in giant craters in the Scotia in comparison with other pockmarks in different areas of the world.

Using a GIS-Based Semi-Automated Toolbox^63^ for characterization of craters shows that pit craters and giant pockmarks) in Scan Basin differ significantly from with pockmarks reported from North Sea^34^, Barents Sea^35,36^ and other regions^,^ (Fig. 7). In particular, pit craters in Scan Basin are characterized by high Vertical Relief (VR) and large Area in comparison with seafloor features observed elsewhere on the modern seafloor (Fig. 7). They are extraordinary, and demand a mechanism for overburden collapse.

**Fig. 8:**
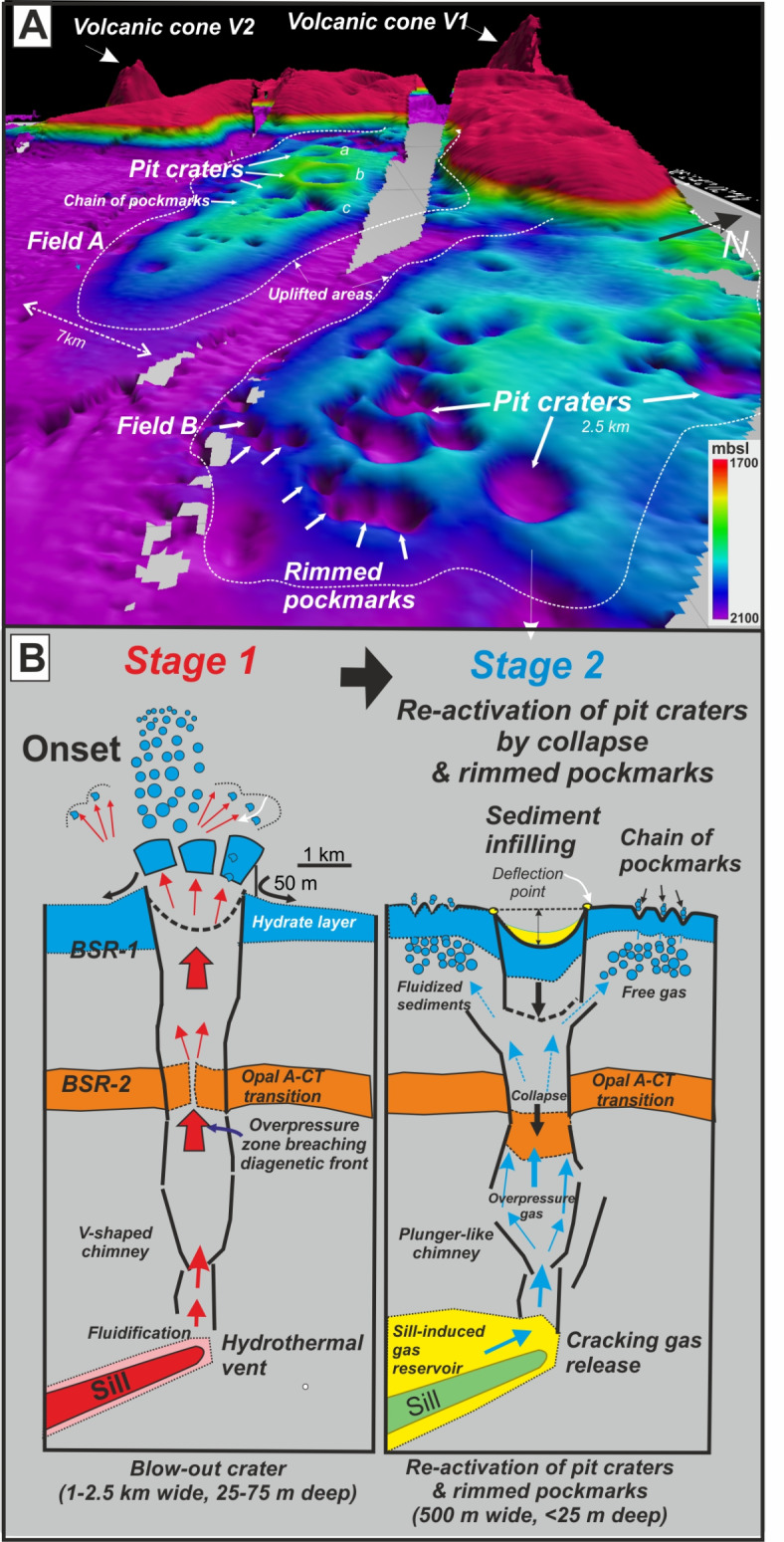
(**A**) View of the field of pit craters from the west showing the morphology of pit craters and rimmed pockmarks. V1 and V2 are volcanic cones related to magma source feeding the induced sill intrusions beneath the pit crater field (dashed lines). (**B**) Two-stage model for explaining the dimensions and shape of the pit craters and rimmed pockmarks: Stage 1—Formation of giant-blow-out craters by hot, buoyant hydrothermal fluids forming V-shaped chimneys. Stage 2—Release of thermogenic methane from the sill-induced reservoir migrating through the former conduit. As increasing the gas overpressure from the sill-induced reservoir then upward flow of gas is triggering through the breaching opal A-CT transition to subseafloor. As decreasing the gas overpressure in the conduit, then the pit crater collapses forcing to the shallow gas to migrate to seafloor forming rings and chains of `standard´ pockmarks distributed radially around the pit crater as observed on seafloor topography in (**A**). This mechanism would explain the successive reactivations of the pit craters caused by episodes of collapse, sedimentary infilling and overpressure peaks as observed in seismic images from 0.9 Ma to present (Fig. 5).

Based on available bathymetric and seismic images, we propose a model for the formation of pit craters and their associated pockmark fields in Scan Basin (Fig. 8). The model considers seafloor features and deep-rooted sills as components of the HTVCs, and where episodic fluid migration has led to crater formation, repeated crater collapse and multiple intervals of partial sediment infilling.

In a first stage (1 in Fig. 8), hot (T∼350º C) and buoyant fluids (including gas) formed and flowed through cracks that define the wide subsurface chimneys connected sills to the seafloor. Such fluid migration breached an interface between consolidated underlying hard sedimentary rock, including possibly an opal A/opal CT horizon, and overlying non-lithified sediment. This is now wonderfully expressed in seismic profiles, where the prominent BSR-2 drops downward across VCZs and the where the V-shaped chimneys begin in the stratigraphic column (Fig. 5B).

Upward advection of hot fluids dissociates gas hydrate in shallow sediment below subseafloor (Fig. 8B). Gas release from rising fluids combined with dissociation of gas hydrates lead to blow out craters similar to those identified as consequence of cryovolcanic process on icy planets, planetoids and polar areas of the Earth, for example Yamal Crater in Russia^64^.

Once gas escapes lithified sediments, as suggested by the breaching of Reflector -c in seismic profiles (Fig. 5B), the second stage involves release of over-pressured gas through unlithified sediment to the seafloor. Sill intrusions should produce thermogenic CH_4_ through the cracking of organic matter in surrounding sediments^23,24^ (Fig. 8B). When the pressure of methane within pore space exceeds lithostatic pressure, particularly along axes of forced-folds, fracturing might take place, forming observed conduits towards the seafloor^53^.

Successive reactivation of fluid flow conduits and craters manifest on seismic profiles as recurring units of sedimentary infill within V-shaped craters from 0.9 Ma to present (Fig. 5D; Table 1). One might suggest variations in sediment accumulation over the late Pleistocene and Holocene^29^. It might alternatively reflect repeated collapses of a geochemical horizon (such as the base of gas hydrate) which might act as a `hydraulic pressure control valve´ by not allowing fluid flow after a major increase in release of over-pressured gas and subsequent drop in fluid pressure. An idea is that, when overpressure fluid flow ceased, and craters collapsed, residual overpressure of gas escaped at the sides of pit craters, leading to formation of rimmed pockmarks surrounding the pit craters (stage 2 in Fig. 8).

Fracturing of the host rock during magmatic intrusion can influence the nature of fluid flow and mineralization in the immediate vicinity of magmatic intrusions, where temperatures exceed the critical temperature of water above 450 ºC and pressures above 498 bars^60^. In oceanic basins, the water depth necessary to water to be supercritical if contact with a volcanic sill at temperatures higher than 450 ºC is about 2940 m^61^. The confined reservoirs above sills are located at 1.5–2 km below seafloor and 2–3 km water depths (Fig. 6). At this temperature and pressure conditions, we suggest that fluids from the confined reservoir might be composed of supercritical fluids ScriW (Fig. 6). This characteristic infers to the fluid migrating upwards, the capacity to breach up to 1 km thickness of the host-rock, including highly cemented silica diagenetic fronts and hydrate layer (Fig. 8). The formation of supercritical waters (ScriW) in such buried hydrothermal systems^60^ open the possibility of: (i) transporting thermogenic hydrocarbon fluids generated by cracking above sills over long distances, and (ii) initiating serpentinization processes at temperatures of 200ºC to 500ºC where peridotites in deep crust/mantle are transformed to serpentinite minerals with production of abiotic methane. The serpentinization of the oceanic crust^65^ by the circulation of these hydrothermal buried reservoirs of ScriW fluids open an important via for generating abiotic methane in other extraterrestrial bodies.

### Implications toward understanding ancient HTVC systems and past carbon cycling

Numerous pit craters and pockmarks characterize the seafloor of Scan Basin, and seismic profiles strongly suggest vertical fluid conduits connect these bathymetric features to magmatic sills that intruded underlying sedimentary strata at > 1.5 km depth below the modern seabed. In multiple respects, including dimensions of craters and conduits, Scan Basin appears to be a modern example of a submarine region with HTVCs.

This important discovery immediately raises two issues facing interpretations of similar systems in the geological record. First, while the craters and pockmarks in Scan Basin lie at the modern seafloor, they represent features that have evolved over at least 0.9 Ma -- they do not indicate sudden widespread fluid venting initiated in the Holocene or latest Pleistocene. Second, while the modern craters coincide with massive injection of^13^C depleted carbon to the atmosphere and ocean (and a developing global negative CIE), the latter is universally ascribed to anthropogenic extraction and combustion of fossil fuels. The two phenomena correspond in time but are not causally related. So, how and why can craters of HTVC systems in ancient strata appear linked to geologically short-term negative CIEs?

A good answer lies in classic stratigraphic principles: the age of crater fill does not give the age and history of crater formation. For buried pit craters in the North Sea, recently drilled by IODP Expedition 396, and which are filled with sediment deposited during the PETM^3,4^, this idea is straightforward to explain with our current understanding of the PETM and existing data, including our observations from Scan Basin. Almost with certainty, abrupt warming during the PETM amplified the global hydrological cycle, increasing sediment discharge from many continental margins^66^, including in the North Sea^67^. The lone buried pit crater recently drilled in the North Sea at Sites U1567 and U1568 is filled with sediment deposited during the PETM, but importantly the sediment contains abundant terrestrial material and not from deeper stratigraphic depths^68^. We suggest that the North Atlantic seafloor near the PETM looked like the modern seafloor in Scan Basin with long-lived craters linked to magmatic sills. In the case of North Atlantic and during the PETM, however, numerous seafloor craters were rapidly filled because of extraordinary climate change.

## Conclusions

Here we document a Hydrothermal Vent Complex (HTVC) formed by intrusion of magmatic sills into the sedimentary basin and connected throughout vertical fluid escape pipes to seafloor pit craters in the Scotia Sea, off Antarctica. Our key findings are as follows:

- The timing of sill intrusion based on seismic stratigraphic relationships of forced folds reveals that sills were intruded during, at least, two diachronous sub-volcanic pulses. An oldest pulse ca. 14.9–12.6 Ma (Middle Miocene) associated with deep intrusions of stacked composite sills along the deep Scan Basin. A later magmatic pulse took place at ca. 0.9 Ma associated with vulcanism developed along the Discovery Bank that reactivated and triggered fluid venting from the former reservoir. This last pulse was responsible to generate the huge field of giant seabed pit craters identified by multibeam bathymetry at the seafloor.
- The spatial association of the sills, pipes, pit craters and pockmarks suggests re-use of the fluid migration pathway(s) extended for over ca. 15 Ma in this HTVC.
- We document with seismic images the re-activation of the pit craters associated to the last magma pulse (0.9 Ma) during the Pleistocene times at 0.78 Ma, 0.43 Ma and 0.14. Ma.
- Besides the absence of exploration of present seafloor activity, and based on their present morphological expression on seafloor, we support that the giant pit craters formed above sill complex A are still active or, at least, latent.
- We have used a GIS-Based Semi-Automated Toolbox for characterization of the seabed pit craters related to the last magma pulse identified in the Scotia Sea in comparison with `standard size´ pockmarks reported in Barents Sea, North Sea, Irish Sea and other regions. This shows that Antarctica pit craters are significantly different that of the most of the `shallow waters´ hydrocarbon basins.
- Based on available bathymetry and seismic images, we propose a model for the formation of pit craters and associated pockmark fields in Scan Basin. Our model is based on the two-stages fluid venting sourced from the sills intrusions: (i) the first stage, after the sill intrusion, gas release from rising fluids combined with dissociation of gas led to form blow-out craters. This high temperature fluid flows allowed to breach and collapse the highly-cemented opal silica horizon; (ii) Once gas could escape lithified sediment, the second stage involves release of over-pressured gas through unlithified sediment to the seafloor. Sill intrusions should produce thermogenic CH_4_ through the cracking of organic matter in surrounding sediments^23,24^. When the pressure of methane within pore space exceeds lithostatic pressure, particularly along axes of forced-folds, fracturing might take place, forming observed conduits towards the seafloor. We suggest that when overpressure fluid flow ceased, and pit craters collapse, residual overpressure of gas escaped at the sides of pit craters, leading to the formation of rimmed chains of pockmarks as identified on multibeam bathymetry. Successive reactivation of fluid flow conduits and later pit crater collapses manifests on seismic profiles as recurring units of sedimentary infill within the V-shaped craters from 0.9 Ma to present.
- In contrast to previous studies, where the likely geologically instantaneous growth of a forced fold and release occurs in response to the intrusion of an isolated sill the evolution of the HTVC system analyzed here occurred over ca. 15 Ma and is associated with the emplacement of multiple, overlapping and stacked sheet intrusions.
- This study highlights: (i) that the intrusion of magma, and associated fluid venting within sedimentary basins may occur over more prolonged time periods than previously considered and (ii) that fluid release from magma intrusions into deep sedimentary basins are long-lived system.
- We here show that the imprints of outburst releases of potential supercritical thermogenic gases from sill-induced deep reservoirs are similar to the mechanism described to explain catastrophic episodes of global warming during the geological history of the Earth. Therefore, we consider that this finding of pit craters associated with the generation of vast and long-term hydrothermal-hydrocarbon sub-seafloor systems under the Antarctica seafloor as a recent analogue of a potential process argued for triggering global warming events.

## Methods

### Multichannel seismic reflection profiles

This study uses broad network of zero-phase, time migrated, 2-D multi-channel seismic (MCS) reflections surveys acquired during the Scan cruises onboard the R/V Hespérides from 1997 to 2014 in the framework of the SCAN (SCotia-ANtarctica plate boundary project) of the Spanish National Program for Antarctic^26–31^. These data image to a depth of 8–9 s two-way time (TWT) are displayed with SEG positive polarity i.e., downward increase in acoustic impedance correspond to a positive reflection whereas a decrease corresponds to a negative reflection. We used Kingdom Suite to display seismic images. The SCAN MCS profiles were obtained with a tuned array of 5 BOLT airguns with a total volume of 22.14 l and a 96-channel streamer with a length of 2.4 km. The shot interval was 50 m and the data were recorded using a GEOMETRIC Strata Visor1 digital system at a sampling record of 2-ms interval and 12-s length. The data were processed with a standard sequence, including migration using a DISCO/ FOCUS system. More information of SCAN seismic profiles can be found in Pérez et al.^26^. Of these MCS profiles, SCAN2004_SC07 and SCAN2004_SC10 were used to cross respectively over International Ocean Discovery Program (IODP) Expedition drilling Sites U1536 and U1537 carried out in the Scotia Sea in 2019^25^ .

To assist the correlation with SCAN MCS profiles, the profile IT89AW39A of the Antarctic Seismic Data Library System (https://sdls.ogs.trieste.it/) have also been used. The seismic data were interpreted using the IHS Kingdom software.

Regional background bathymetry from Fig. 1 has been generated using GeoMapApp 3.7.4 (August 2024) (www.geomapapp.org)^6^. GeoMapApp is designed for both public and academic use, and is licensed under a Creative Commons Attribution International License.

The swath data for the other figures were obtained with a KONSBERG EM 12 system during SCAN1997, SCAN2004, SCAN2008 and SCAN2013 cruises and post-processes with the NEPTUNE software and FLEDERMAUS for visualization.

### Automatic morphometric characterization

We use a new semi-automated tool box methodology developed on ArcGIS for the morphometric characterization of craters and pockmarks^63^.The morphometric characterization of vast numbers of sub-circular depressions allows an unprecedented statistical analysis of their morphology.

## Electronic supplementary material

Below is the link to the electronic supplementary material.

Supplementary Material 1

Supplementary Material 2

Supplementary Material 3

## Data Availability

Contact to Luis Somoza (CN IGME-CSIC) (l.somoza@igme.es) to request data provided within the manuscript or supplementary files from this study.
