# Supplementary figures and images for: Giant pit craters on the modern seafloor above magma-induced hydrothermal vent complexes of Scotia Sea, offshore Antarctica
Source: Sci Rep. 2025 Jan 24;15:3139. doi: 10.1038/s41598-025-85899-y (PMC11760963; doi:10.1038/s41598-025-85899-y)

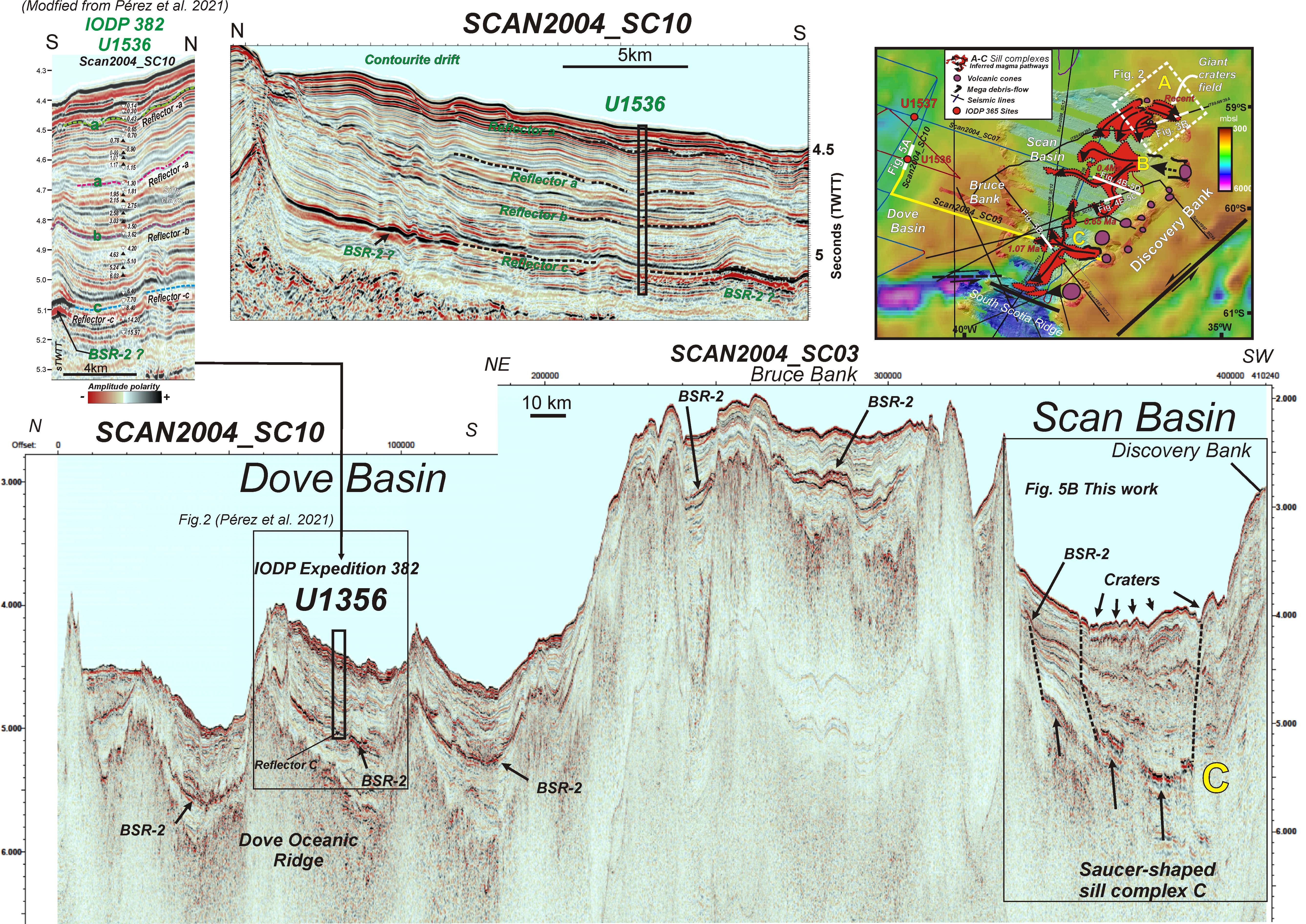

Supplement: Supplementary file 1 — Supplementary Material 1 [file 41598_2025_85899_MOESM1_ESM.jpg]

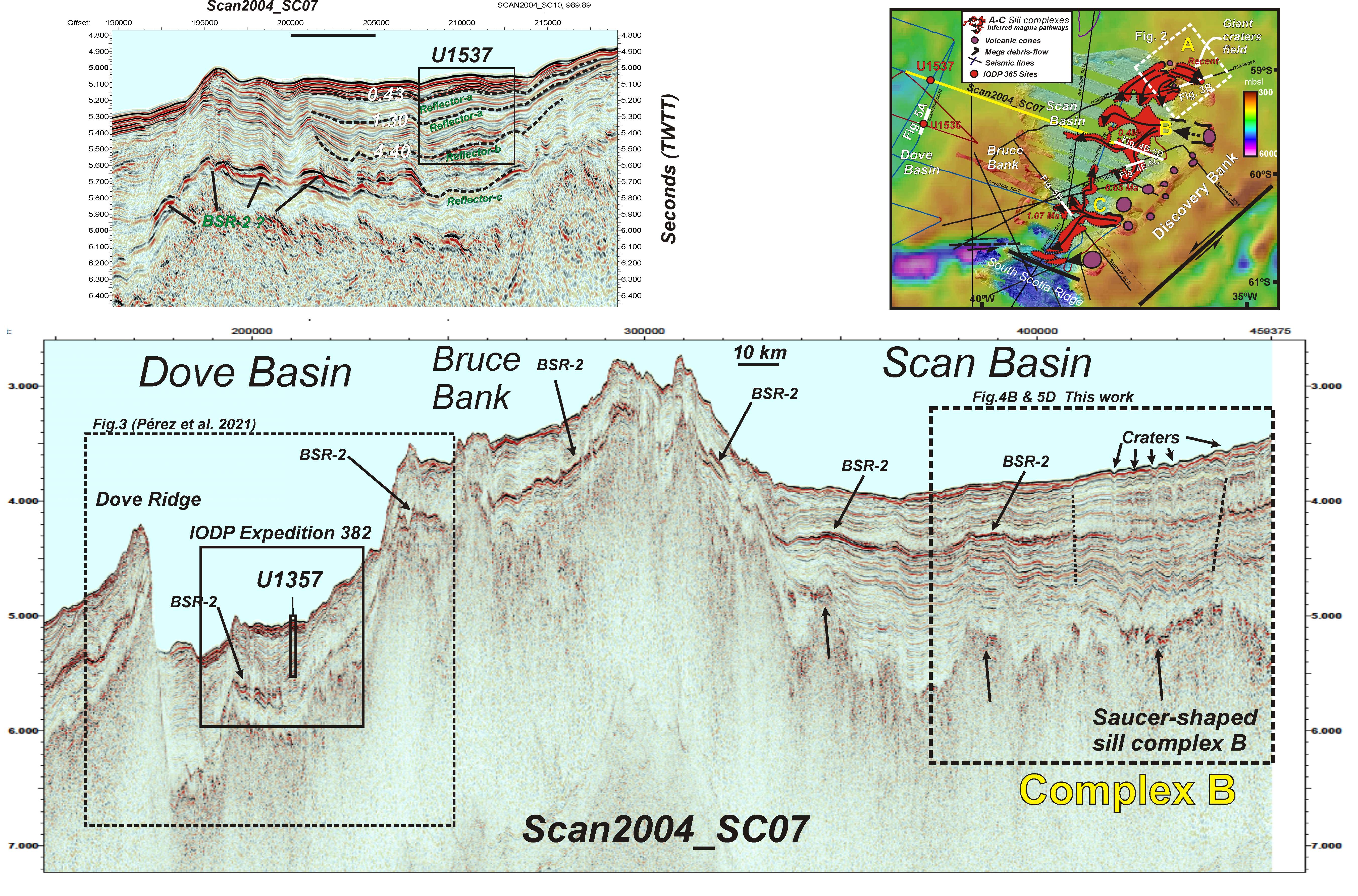

Supplement: Supplementary file 2 — Supplementary Material 2 [file 41598_2025_85899_MOESM2_ESM.jpg]
