# Supplementary material for: Giant pit craters on the modern seafloor above magma-induced hydrothermal vent complexes of Scotia Sea, offshore Antarctica
Source: Sci Rep. 2025 Jan 24;15:3139. doi: 10.1038/s41598-025-85899-y (PMC11760963; doi:10.1038/s41598-025-85899-y)
Supplement: Supplementary file 3 — Supplementary Material 3 [file 41598_2025_85899_MOESM3_ESM.pdf]

Volcanic and subvolcanic samples in the Discovery Bank close to the craters field

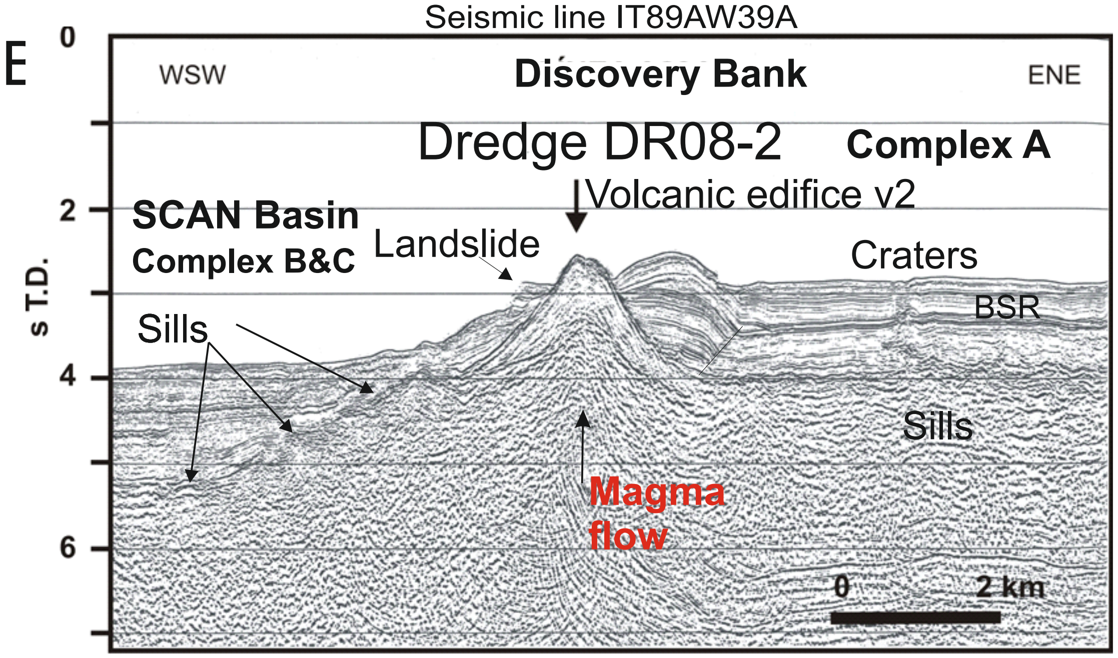

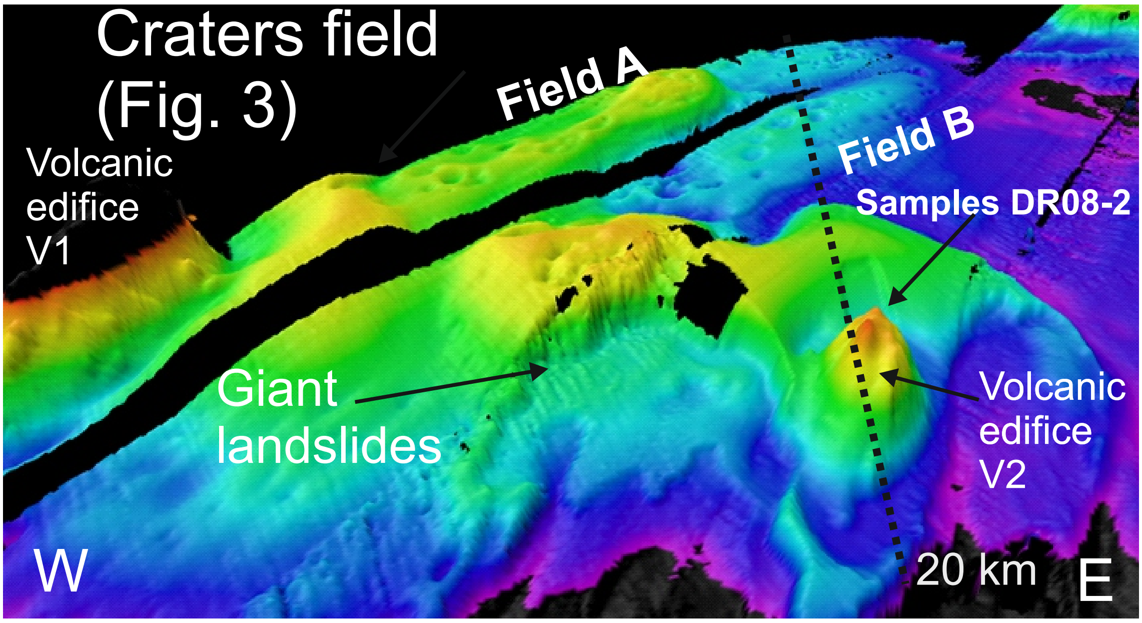

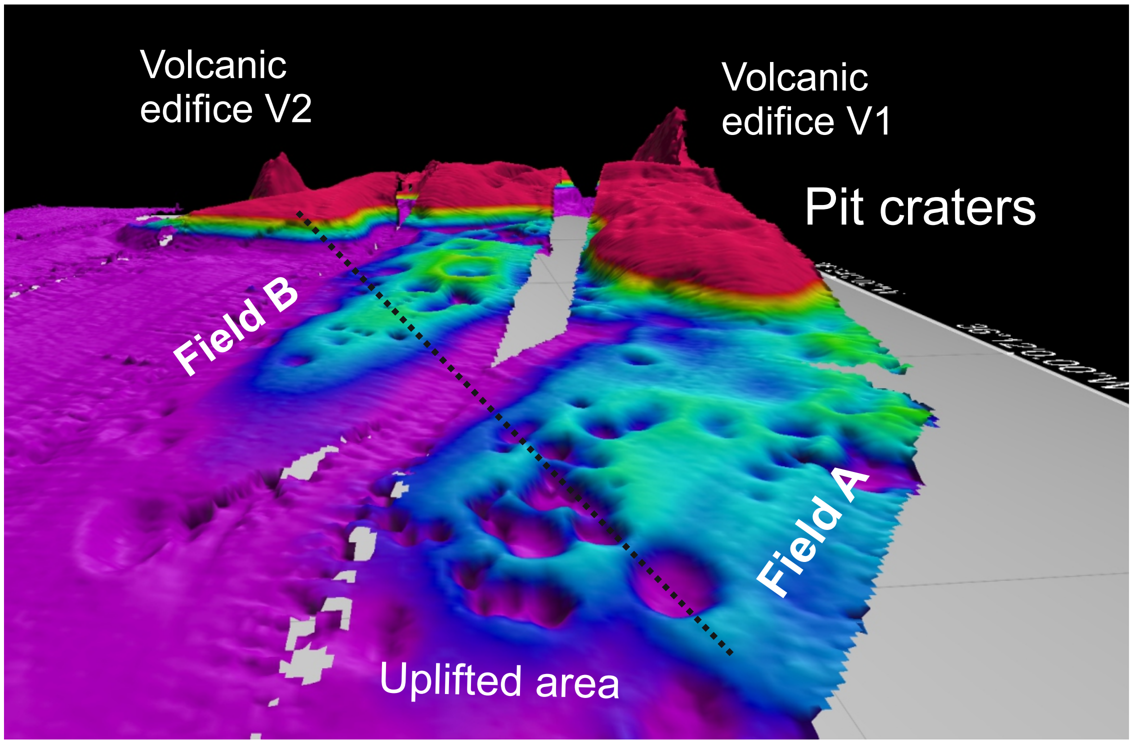

Volcanic and subvolcanic samples in the Discovery Bank close to the craters field

Samples from dredge DR08-2 (Volcanic edifice V2)

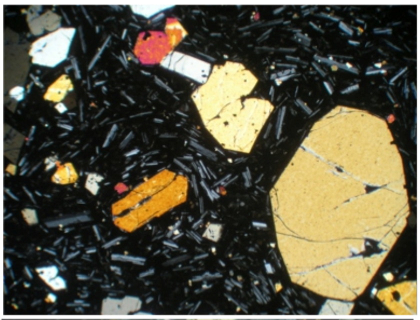

Fresh olivine basalts (OIB)

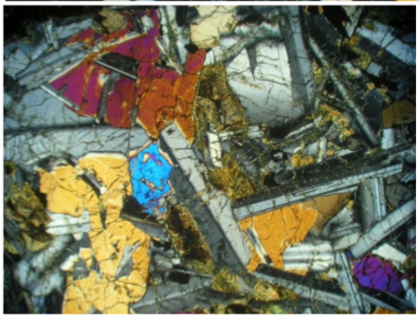

Altered olivinic dolerite (back-arc)

Description of samples

Samples were dredges from the top of different seamounts related to Dove and Scan Basin during the SCAN-2004 and SCAN-2008 cruises.

In order to avoid sampling ice-draft samples we mainly dredged the top and the step slopes of the seamounts. Even though it might have been some doubts over origin of samples, in-situ samples showed clearly evidence of fresh fracture zones when they were picked up aboard. Two main types of rocks were dredged from these seamounts: **volcanic rocks, basalts and trachyandesites,and greenschist granulite and amphibolites**.

In the seamounts eastwards Scan basin, the most distinct features were the greenschist amphibolites. Thorium, Ta, Tb and Yb contents and their ratios plotted on discriminating diagrams evidence that samples from seamounts eastwards Scan Basin are comparable to a **continental margin and supra-subduction magmatism** (Puga et al., 2004; 2008).

References

E.Puga, A.M.Álvarez-Valero, J.Galindo-Zaldívar, F.Bohoyo, A.Maldonado, A.Schrider, E.Suriñach, F.J.González, F.Lobo, L.Somoza, A.Díaz de Federico. The Dove Basin (Scotia Sea, Antarctica): Geodynamic setting of its volcanism, magnetic anomalies and K/Ar dating 1 Symp. Antarctic Climate Evolution. Granada, Sept. 2009

E. Puga, A.M. Álvarez-Valero, J. Galindo-Zaldívar, F. Bohoyo, A. Maldonado, F.J. González, L. Somoza, F. Lobo. (2009). Petrologic, geochemical and genetic conditions of the volcanism generating the Dove Ridge (Scotia Sea, Antarctica). 1 Symp. Antarctic Climate Evolution. Granada, Sept. 2009
